# Per- and Polyfluoroalkyl Substances (PFASs) and Their Potential Effects on Female Reproductive Diseases

**DOI:** 10.3390/toxics12080539

**Published:** 2024-07-26

**Authors:** Yuqing Yi, Yang Feng, Yuechen Shi, Jiaming Xiao, Ming Liu, Ke Wang

**Affiliations:** 1 Department of Clinical Nutrition, Second Affiliated Hospital of Dalian Medical University, Dalian 116027, China; yiyuqing072@163.com (Y.Y.); fy20220322@163.com (Y.F.); 13624107357@163.com (Y.S.); xjm155333@163.com (J.X.); liuming9821@163.com (M.L.); 2 Department of Nutrition and Food Hygiene, School of Public Health, Dalian Medical University, Dalian 116044, China

**Keywords:** PFASs, emerging persistent pollutants, female reproductive diseases, polycystic ovary syndrome, endometriosis, primary ovarian insufficiency, diminished ovarian reserve

## Abstract

Per- and polyfluoroalkyl substances (PFASs) are a class of anthropogenic organic compounds widely present in the natural and human living environments. These emerging persistent pollutants can enter the human body through multiple channels, posing risks to human health. In particular, exposure to PFASs in women may cause a series of reproductive health hazards and infertility. Based on a review of the existing literature, this study preliminarily summarizes the effects of PFAS exposure on the occurrence and development of female reproductive endocrine diseases, such as polycystic ovary syndrome (PCOS), endometriosis, primary ovarian insufficiency (POI), and diminished ovarian reserve (DOR). Furthermore, we outline the relevant mechanisms through which PFASs interfere with the physiological function of the female ovary and finally highlight the role played by nutrients in reducing the reproductive health hazards caused by PFASs. It is worth noting that the physiological mechanisms of PFASs in the above diseases are still unclear. Therefore, it is necessary to further study the molecular mechanisms of PFASs in female reproductive diseases and the role of nutrients in this process.

## 1. Introduction

**Figure 1:**
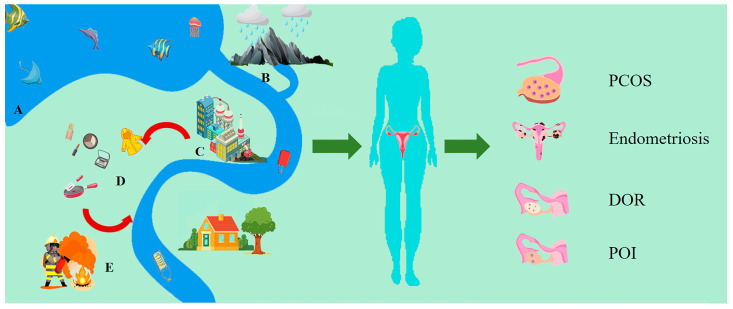
PFASs harm the female reproductive system through various routes of exposure. A: Seafood. B: Surface water. C: Factory. D: Water-repellent clothing, cosmetic, non-stick cookware. E: Fire-fighting foam.

Per- and polyfluoroalkyl substances (PFASs) are known for their distinctive chemical structures, featuring partially or completely fluorinated methyl or methylene carbon atoms in carbon chains of varied lengths (-C_n_F_2n+1_-). Perfluoroalkyl substances are formed when all hydrogen atoms in the carbon chain of chemicals are replaced by fluorine atoms [1]. These compounds are highly resistant to environmental and biological degradation due to their chemical stability, heat resistance, hydrophobicity, and lipophilicity owing to the exceptional strength of their carbon–fluorine (C–F) bonds. The above characteristics lead to extremely durable and useful surfactants and polymers [2]. According to the recognized PFAS classification system prepared by Buck et al., PFASs can be divided into two categories, among which long-chain PFASs (PFCAs (perfluoroalkyl carboxylic acids) with eight carbons and greater; and PFSAs (perfluoroalkane sulfonates) with six carbons and greater) include some of the most well-studied chemicals, such as perfluorooctane sulfonate (PFOS) and perfluorooctanoic acid (PFOA) [2]. Depending on the number of CF2 moieties, PFASs are classified as ultra-short-chain or short-chain PFASs when the numbers of perfluorocarbon atoms are 2–3 and 4–7, respectively [3]. Consequently, PFAS substances have been extensively used in a wide array of industrial and consumer chemical products since the 1940s, spanning over 100 sectors. Industrial applications include polytetrafluoroethylene (PTFE) production, and they can also be used in the metal plating industry, photo imaging industry, and semi-conductor industry. PFASs are also used in large-scale consumer goods, including food packaging, cosmetics, water-repellent clothing, textiles, non-stick cookware, cleaning agents, papermaking, fire-fighting foam, and medical products [4,5]. Due to their ubiquitous applications globally, PFASs have been detected in environmental media such as surface water, groundwater, seawater, soil, sediment, and the atmosphere, as well as in humans and wildlife, which is most concerning [6,7,8,9]. Current evidence indicates that the exposure of humans to PFASs mainly occurs through the consumption of contaminated water and seafood, the inhalation of indoor air and dust particles, hand-to-mouth contact, and skin absorption [5,10]. Higher levels of PFOA (lower bound/upper bound (LB/UB) mean = 0.18/0.9 µg/kg) and PFOS (LB/UB mean = 2.08/2.59 µg/kg) have also been detected in fish and other seafood [11]. The European Food Safety Authority (ESFA) CONTAM panel reported that over 86% of PFAS exposure in the diet may be attributed to seafood consumption [12] (Figure 1). Among the vast array of PFASs, there are a number of long-chain PFASs that have been subjected to regulatory scrutiny and discontinued or regulated due to their environmental toxicity and bioaccumulation [13,14]. Moreover, additional long-chain PFAS substances that have raised concerns, specifically PFOA and perfluorohexane sulfonate (PFHxS), along with their salts, were phased out of global production in 2021 and 2022 [15,16]. Accordingly, manufacturers have begun to undertake commercial production using short-chain PFASs, ultra-short-chain PFASs and new alternatives [3]. Compared to the PFASs used in the past, short-chain PFASs are generally less bio-accumulative, but they show higher environmental persistence and long-distance mobility. Continuous exposure to such compounds can adversely affect organisms in the long term [17]. 

Previous studies have demonstrated the connections between PFAS exposure and increased odds of cancer, kidney disease, altered immune function, liver disease, lipid and insulin dysregulation [18]. Although many biological systems are adversely affected by PFASs, in this review, we focus on their relevant effects on female reproductive health. Epidemiological studies indicate that infertility, a critical issue in women’s reproductive health, now affects approximately 48 million couples and 186 million individuals globally, highlighting its status as a significant public health concern. Disease-related infertility, an important contributor affecting spontaneous conception, has attracted a wide range of attention [19]. PCOS, endometriosis, POI, and DOR are all factors contributing to female infertility. Studies suggest that certain long-chain PFASs could exacerbate these conditions and increase the risk of them arising [20,21]. In addition, results from animals and epidemiological studies have corroborated that short-chain PFASs such as hexafluoropropylene oxide dimer acid (GenX) and F-53B (6:2 chlorinated polyfluoroalkyl ether sulfonate [6:2 Cl-PFAES] and 8:2 Cl-PFAES) are becoming the dominant perfluorinated contaminants globally, and these also exhibit reproductive toxicity [22,23]. GenX exhibits a stronger affinity for the estrogen receptor (ER), compared to PFOA, and F-53B has been shown to reduce the spawning output in zebrafish [24].

The mechanisms by which female reproductive system diseases are affected by PFASs are often complex. First, kisspeptin is an influential neuroendocrine peptide in the hypothalamus that regulates the secretion of gonadotropin-releasing hormones (GnRHs), the release of which initiates a series of responses that trigger ovulation. Exposure to PFASs inhibits pro-peptide expression in areas of the brain such as the anteroventral periventricular nucleus (AVPV), which is essential for the production of the luteinizing hormone (LH) surge required for ovulation. This inhibition leads to reduced levels of GnRH and LH, ultimately interfering with the normal ovulatory cycle and potentially leading to ovulation failure [25,26]. Additionally, PFASs can influence the expression of crucial steroidogenic enzymes and either mimic or obstruct the actions of hormone receptors, thereby disrupting hormone-signaling pathways. This interference with normal hormone functions can lead to significant repercussions for reproductive health, altering fertility and menstrual cycles [27,28,29,30]. Oxidative stress is a state wherein there is an imbalance between oxidation in cells and tissues and the antioxidant systems, and it has been proven to play a key role in the pathogenesis of reduced female fertility. This imbalance can lead to various reproductive system disorders, such as endometriosis, PCOS, and unexplained infertility [31]. Oxidative stress can also lead to chronic anovulation and infertility in women by exacerbating insulin resistance and impairing oocyte development and quality [32]. Furthermore, numerous studies have shown that PPARs (peroxisome proliferators-activated receptors) play a role in normal reproductive and developmental functions, and that exposure to endogenous or exogenous compounds that abnormally modulate PPARs may lead to physiological dysfunction of the reproductive system [33]. The sustained activation of ovarian PPARs by PFAS exposure may disrupt oocyte meiosis while increasing lipid accumulation in the ovary and decreasing oocyte viability [26,34].

In this review, we summarize the associations between human PFAS exposure and four reproductive health outcomes in women that have been studied to date, including PCOS and endometriosis, as well as POI and DOR. We also outline the potential mechanisms through which PFASs may affect female ovaries, and, finally, we highlight the role of nutrition in mitigating the adverse effects of PFASs on ovarian cells.

## 2. Search Strategy

To explore the potential connection between PFAS exposure and female reproductive health, this review mainly focuses on the prevalence and impact of PFASs in relation to PCOS, endometriosis, POI, and DOR. Emphasis was placed on the analysis of epidemiological data and the study of molecular mechanisms affecting ovary function with PFASs.

Additionally, databases and scientific studies were explored using PubMed. The search terms included “Per- and poly-fluoroalkyl substances”, “Endocrine-disrupting chemicals”, “PFASs”, “PFOA”, “PFOS”, “short-chain PFASs”, “PFAS alternatives”, “Polycystic ovary syndrome”, “Endometriosis”, “Premature ovarian insufficiency”, and “Diminished ovarian reserve”.

Data inclusion and exclusion: We included only cohort and case–control studies that assessed the association between PFAS and PCOS, endometriosis, POI, DOR, and studies on the mechanisms by which PFASs may affect diseases of the female reproductive system, and we excluded non-original reports, review articles, conference abstracts, editorials, commentaries, experimental or pilot studies, case reports and case series, as well as studies that did not measure PFAS exposure. The findings were restricted to those published in English.

## 3. Human Exposure to PFASs and Female Reproductive Disorders

### 3.1. Polycystic Ovary Syndrome

Polycystic ovary syndrome is a heterogeneous disorder that affects 6–20% of premenopausal women, which makes this syndrome the most common endocrine metabolic disorder in women of reproductive age and accounts for about 80% of women with anovulatory infertility [35,36]. Recent studies have indicated that a range of environmental factors could potentially play a significant role in the initiation or exacerbation of PCOS. These factors are thought to interact with genetic predispositions, potentially triggering or worsening the symptoms associated with this endocrine disorder [37,38]. For example, studies have shown that there is a positive correlation between serum bisphenol A (BPA) levels and serum testosterone (T) levels in PCOS women, compared to healthy women [39]. Vagi et al. enrolled 52 patients with PCOS and 50 controls from an urban academic medical center in California, USA, to explore potential associations between polycystic ovary syndrome and perfluorinated compounds, polychlorinated biphenyls (PCBs), BPA, and organochlorine pesticides. They found that participants with PFOA and PFOS serum concentrations in the highest tertile were 6.9-fold and 5.8-fold more likely to develop PCOS compared to controls, respectively. This is the first evidence of a link between serum PFAS concentrations and PCOS [40]. Heffernan et al. recruited 30 PCOS women and 29 controls in the UK, collecting their serum and follicular fluid samples. Serum PFOS was higher in PCOS patients than in controls (geometric mean 3.9 vs. 3.1 ng/mL, *p* < 0.05), while the geometric mean follicular fluid concentration did not differ significantly between the groups [41]. In another case–control study of endocrine disruptors and female infertility, Zhan et al. studied women from the Shanghai, Shandong, and Zhejiang provinces, China. They measured the serum levels of nine legacy PFASs, six PFAS isomers, three emerging PFAS alternatives, and six short-chain PFASs in 366 PCOS cases with 577 controls. PCOS risks were considerably enhanced by higher concentrations of PFASs in serum. In these PFAS families, 6:2 chlorinated polyfluorinated ether sulfonate (6:2 Cl-PFESA), hexafluoropropylene oxide dimer acids (HFPO-DA), perfluorododecanoic acid (PFDoA), and ∑3,4,5 m-PFOS may be available as the primary contributors. They also observed that the joint effects of PFAS mixtures are more pronounced in overweight/obese women [42]. However, the connections between PFAS exposure, BMI, and the incidence of PCOS remain unclear. Further research is essential to elucidate these relationships more comprehensively [43]. Li et al. tested 218 healthy women, as well as 73 women with PCOS, for 12 PFASs in follicular fluid samples and sex hormone concentrations in serum in Shanghai, China. Specifically, the levels of LH and T were notably higher in the PCOS group compared to the healthy controls (*p* < 0.001). Moreover, the study revealed a significant positive potential association between PFOA exposure and PCOS in all participants (OR (95% CI): 1.74 (1.17, 2.64), *p* = 0.007). Additionally, they undertook a mediated effect analysis to explore the pathways through which PFOA might influence the development of PCOS. The analysis indicated that PFOA is not mediated but directly involved in the pathogenesis of PCOS through T [44].

### 3.2. Endometriosis

Endometriosis is a complex, estrogen-dependent condition characterized by chronic inflammation and significant pain, affecting between 6% and 10% of women in their reproductive years across the globe. This chronic gynecological disorder can significantly impact a woman’s quality of life. It is also a well-recognized reason for infertility, contributing to the challenges faced by many trying to conceive. Alarmingly, studies have shown that up to 50% of women who are experiencing infertility may have endometriosis, highlighting its profound impact on reproductive health [45,46]. Numerous studies have explored the connection between endometriosis and levels of PFASs in the body. Buck Louis et al. recruited two samples, one of which consisted of 495 women who underwent laparoscopic or open-heart surgery in the Salt Lake City or San Francisco area, and the other of which included 131 women who were residence-matched. The authors first reported that PFOA and perfluorononanoic acid (PFNA) in the operative sample were associated with increased odds of endometriosis when adjusting for confounding factors [47]. Campbell et al. collected data (*n* = 753) on doctor-diagnosed endometriosis and those with serum measurements of PFASs from the 2003–2004 to the 2005–2006 National Health and Nutrition Examination Surveys (NHANES). They observed that women with endometriosis had significantly higher geometric mean levels of serum PFOA (3.48 vs. 2.84 ng/mL), PFOS (16.28 vs. 13.36 ng/mL), and PFNA (1.00 vs. 0.84 ng/mL) compared with women who did not report endometriosis. However, PFHxS was not significantly associated with an increased risk of endometriosis [48]. Wang et al. also examined PFAS concentrations in women who provided serum samples from the Women’s Hospital Affiliated to Zhejiang University School of Medicine. A total of 157 endometriosis-related infertility cases and 178 women without any reproductive endocrine disease were selected. In the majority of PFASs with a detection rate exceeding 98.5%, the case group consistently exhibited higher concentrations compared to the control group. Elevated levels of perfluorobutanesulfonic acid (PFBS) are statistically significantly correlated with an increased risk of endometriosis, while the possibility of reverse causation is excluded [49]. In a recent case–control study by Ao et al., 240 women diagnosed with endometriosis were compared with 334 normal controls. The findings reveal that the combined impact of a mixture of PFASs is significantly associated with an increased risk of endometriosis, primarily influenced by 1 m-PFOS, a specific PFAS compound. At the same time, each unit increase in the PFAS mixture was found to account for a 24% increase in the prevalence rate of endometriosis [50].

### 3.3. Premature Ovarian Insufficiency

POI is an endocrine disease that occurs in women before the age of 40 years and is characterized by a lack of ovarian sex hormones and a decline in ovarian reserve function, which affects at least 1% of females [51,52]. The underlying causes of POI are not fully understood. However, potential risk factors for these conditions include genetic predispositions, autoimmune disorders, medical treatments, infections, environmental influences, and other factors [53,54,55]. Prior research has shown that exposure to polychlorinated biphenyls (PCBs), PFASs, and phthalates can increase the overall risk of ovarian aging (a natural physiologic aging process in which the number and quality of oocytes or follicular pools decline), leading to declining fertility, earlier age at menopause, the diminishing of ovarian reserves, and premature ovary insufficiency [56,57]. In a Chinese case–control study, Zhang et al. measured the concentrations of nine different PFASs and hormones in blood samples from 120 patients diagnosed with POI alongside 120 control subjects in Shanghai. They observed that in patients with POI, the median levels for PFOA [11.1 (7.60 to 14.45) ng/mL], PFOS [8.18 (5.50 to 13.51) ng/mL], and PFHxS [0.38 (0.29 to 0.67) ng/mL] were all significantly higher compared to the control group’s levels of PFOA [8.35 (6.27 to 11.31) ng/mL, *p* < 0.001]; PFOS [6.02 (4.24 to 9.11) ng/mL, *p* < 0.001]; and PFHxS [0.29 (0.22 to 0.37) ng/mL, *p* = 0.001]. They also found that in POI cases, the levels of exposure to PFOS and PFHxS were positively correlated with concentrations of follicle-stimulating hormone (FSH) and prolactin (PRL), while showing a negative correlation with estradiol (E2) levels. Additionally, exposure to PFOA and PFOS in the control subjects was linked to decreased levels of free triiodothyronine (FT3) and free thyroxine (FT4), alongside an increase in thyroid-stimulating hormone (TSH) concentrations. This illustrated that high exposure to PFASs suppresses ovarian hormone production and impairs follicular development, resulting in the failure of ovarian function [58].

### 3.4. Diminished Ovarian Reserve

DOR is defined as a decrease in the number and quality of oocytes, which is clinically characterized by elevated FSH levels, decreased levels of anti-mullerian hormone (AMH), and a decrease in the antral follicle count (AFC) [59]. The worldwide incidence of DOR among women undergoing fertility treatment is 10% [60]. 

PFASs and other endocrine-disrupting chemicals (EDCs) are a class of chemicals that can be transferred from the bloodstream to the follicular fluid; thus, assessing the levels of such compounds in the follicular fluid can highlight their association with ovarian function [61]. Tian et al. recruited 64 women with DOR and 86 controls in Beijing, China. They collected follicular fluid samples from these participants to study the combined effects of 21 EDCs on DOR. The authors proposed that the likelihood of DOR was strongly correlated with elevated concentrations of perfluorohexanoic acid (PFHxA), indicating that PFHxA was detrimental to the ovarian reserve function [62]. Given the presence of many studies delineating the effects of EDCs on female fertility, the employment of the ovarian sensitivity index (OSI) to evaluate their relationship is of critical importance. This index is related to ovarian reserve markers such as AMH, AFC, and FSH levels [63]. A comprehensive cohort study conducted by Bellavia et al. investigated the association between exposure to EDCs and female fertility. The study encompassed 333 women undergoing Assisted Reproductive Technology (ART) treatments in Sweden and Estonia. A strong negative correlation was observed between the levels of perfluoroundecanoic acid (PFUnDA) and PFOA with the lower OSI, which suggests that these chemicals may interfere with AMH, AFC, and FSH levels in women, triggering the occurrence of DOR [63]. To investigate the effect of the PFOA concentration on embryo quality, Shen et al. examined the concentration of the PFOA in the follicular fluid of 25 DOR patients and 25 normal ovarian reserve (NOR) patients. The results show that the level of PFOA in follicular fluid was higher in the DOR group than in the NOR group (*p* < 0.05). This may be related to the fact that 15α-T promotes the production of estrogen and progesterone and the expression of urocanic acid (UCA) [64].

As summarized in Table 1, recent epidemiological research has increasingly pointed to a link between exposure to PFASs and the development of reproductive health issues such as PCOS, endometriosis, POI, and DOR [21]. However, these studies often face significant limitations. Many rely on retrospective designs that cannot definitively establish causality between PFAS exposure and these specific reproductive conditions, underscoring the urgent need for prospective studies. Additionally, the sample sizes in many of these studies are relatively small, potentially obscuring significant exposure-related differences between affected individuals and control populations. The persistence of PFASs in the human body and their bioaccumulative nature pose further challenges, as their long half-lives mean that short-term exposure assessments may be unrepresentative of long-term body burdens. Most existing studies utilize blood or urine samples, but research into PFAS levels in follicular fluid, which more directly influence oocyte and follicular development, remains scant [65]. As PFASs continue to be pervasive in the environment, the need to understand their impact on female reproductive health becomes increasingly critical.

## 4. Mechanisms of PFASs’ Effects on Female Reproductive Health

### 4.1. PFAS Exposure Suppresses Kisspeptin Signaling and Impairs Reproductive Hormone Regulation

**Figure 2:**
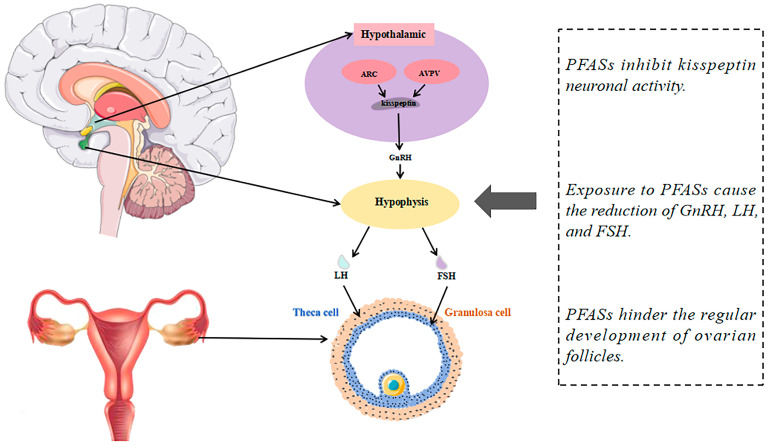
PFASs affect the HPG axis thereby inhibiting sex hormone and follicle production.

Kisspeptin promotes GnRH secretion by activating signaling pathways through its interaction with the Kisspeptin receptor (Kiss1r), thereby playing a critical role in regulating the hypothalamic–pituitary–gonadal (HPG) axis [66]. PFASs interfere with steroid hormones and synthesis and normal oocyte development by modulating the HPG axis (Figure 2). In a previous study, Du et al. used female neonatal rats of the Sprague–Dawley (SD) line subcutaneously injected with PFOA or PFOS at three different doses (0.1, 1, and 10 mg/kg/day) for 5 days (PND1~5 or PND26~30). Compared with the controls, Kiss1 and Kiss1r mRNA expression in the AVPV and hypothalamic arcuate nucleus (ARC) of post-pubertal adult rats was suppressed [67]. In another study, twelve-week-old female mice were divided into four groups, one of which was given PFOS (10 mg/kg) orally for 30 days. In comparison with the controls, reductions in progesterone (P4), GnRH, and LH were observed on the seventh day of PFOS exposure, with decreased levels of E2 and thyroxine (T4) detected on the fourteenth day. These changes may be attributed to significant decreases in the number of Kiss1 cells in the AVPV nucleus, as well as reductions in AVPV-Kiss1 mRNA (*p* < 0.01) and AVPV-Kiss1 protein levels (*p* < 0.05). Furthermore, the authors discovered that PFOS could inhibit the E2-induced surge in serum LH levels, further demonstrating that PFOS exposure directly inhibits E2-activated kisspeptin neurons in the AVPV, thereby suppressing the production of the LH surge [68]. The experiment demonstrated that chronic exposure to PFOS (0.1 mg/kg/day) in adult female rats inhibits the biosynthesis of E2 by reducing the expression of StAR mRNA, which is mediated by the decreased acetylation of histone H3K14, resulting in the decreased transport of cholesterol as an essential precursor for ovarian steroidogenesis [69,70].

### 4.2. Disruption of Steroid Hormone Synthesis Gene Expression and Hormonal Interference by PFASs

The synthesis of steroid hormones involves a complex pathway. Cholesterol, a precursor for steroid hormone synthesis, is transported via the StAR protein to the cytoplasm of follicular membrane cells where it is converted by the enzyme cholesterol side-chain cleavage enzyme (P450scc) to pregnenolone, which is catalyzed by the enzyme 3β-hydroxysteroid dehydrogenase (3β-HSD) to produce progesterone. Progesterone is synthesized into androstenedione by the enzymes 17α-hydroxylase (CYP17A1) and the 17,20-cleaving enzyme-catalyzed synthesis of androstenedione. Cytochrome P450 aromatase (CYP19A1) converts androgens (especially testosterone and androstenedione) to estrogens (estradiol and estrone).

PFASs may interfere with steroid hormone biosynthesis in the female ovary at the molecular level. Kang et al. measured the amounts of E2 and testosterone produced after exposure to PFOA and PFOS (10 pM-10 μM) using human adrenal carcinoma cell H295R. They observed an approximately 2-fold increase in E2 levels when PFOA concentrations were 10 and 100 μM, and an approximately 2-fold increase in E2 levels when the PFOS concentration was only 100 μM; on the other hand, 100 μM of PFOA and PFOS decreased testosterone levels. After assessing the transcript levels of steroidogenic genes using qPCR, they found that PFOA and PFOS exposure increased the gene expression of 3β-HSD and CYP19 by more than 2-fold, and that CYP17 was transcriptionally activated 1.5–2-fold [71]. In contrast, in an experiment involving the in vitro culture of mice follicles, Yang et al. delivered PFOA (100 μg/mL) by oral gavage to 10- to 30-day-old mice. They found that PFOA can lead to decreased levels of estrogen and progesterone and increased testosterone levels compared with vehicle controls by downregulating the expression levels of genes for StAR, 3β-HSD, CYP17A1, and CYP19A1, which may pose a risk of premature ovarian insufficiency [72]. Similar findings were reported for porcine follicular membrane cells and granulosa cells exposed to 1.2 μM PFOS or PFOA. Compared to the control group, they observed the reduced secretion of estradiol, progesterone, and androstenedione, which was attributed to the decreased expression and enzyme activity of CYP17A1 and 17β-hydroxysteroid dehydrogenase (17β-HSD) [73].

### 4.3. PFASs as Estrogen Receptor Agonists and Antagonists Disrupt Estrogen Signaling and Reproductive Effects

Several PFASs can act as agonists or antagonists of the estrogen receptor and exhibit ERα-mediated estrogenic activity [74]. Benninghoff and colleagues utilized Mt Shasta strain juvenile rainbow trout as an animal model. They observed that at the highest tested concentration (1000 ppm), the mixture of PFOA/PFNA/perfluoroundecanoic acid (PFDA)/PFUnDA induced levels of the estrogen-responsive biomarker protein vitellogenin (Vtg) higher than the sum of the individual compounds. This indicates that PFASs induce Vtg expression in vivo, particularly PFOA and PFNA, which exhibit significant estrogen-like activity. Additionally, compounds such as PFOA, PFNA, PFDA, and PFOS can effectively dock with the ERα of various species (humans, mice, and trout) and form hydrogen bonds at the Arg394/398/407 site. This binding behavior suggests that they may disrupt normal estrogen signaling, potentially affecting reproduction and other physiological processes regulated by estrogen [75]. Lending further support, Li et al. conducted a combination of in vitro experiments and computer simulations with MCF-7 BUS human breast adenocarcinoma cells and MVLN cells. Specific PFAS compounds, such as PFHxS and PFOS, were found to act as estrogen receptor agonists, meaning they can mimic estrogen and activate the receptor. In contrast, a positive correlation between short-chain (C-4~C-5) PFASs, such as perfluorobutanoic acid (PFBA), PFBS and perfluoropentanoic acid (PFPeA), and anti-estrogenic activity was observed. PFASs interact with estrogen receptors by binding to their ligand-binding domains, potentially acting as agonists or antagonists, thereby affecting the expressions of estrogen-responsive genes like TFF1 and EGR3 and disrupting normal hormonal signaling [76].

### 4.4. Activation of the PPAR Signaling Pathway and Induction of Oxidative Stress by PFASs

PPARs are a family of nuclear hormone receptors participating in various processes that may affect ovarian function [77]. A study by Zhang et al. revealed that exposure to PFOA in mice significantly reduces the number of primordial and antral follicles. PFOA disrupted mitochondrial physiological functions and the antioxidant system by downregulating key genes associated with mitochondrial complexes and glutathione transferase in mouse ovaries. Meanwhile, PFOA impairs the mitochondrial functionality within granulosa cells, elevates mitochondrial ROS (mtROS) levels in the ovaries, and impedes the proper development of ovarian follicles [78]. Zhang et al. investigated the oral administration of PFOA to adult female mice, resulting in decreased GnRH and LH levels. This phenomenon can be attributed to PFOA’s effect of diminishing the kisspeptin–reproductive endocrine system’s activity, achieved by amplifying the expression of hepatic fibroblast growth factor 21 (FGF21, a peptide hormone that is synthesized by several organs and regulates energy homeostasis) through PPAR-α activation. Consequently, this disruption in the liver–brain reproductive endocrine axis could prolong the inter-estrus phase and, adversely, affect ovulation [79,80]. Table 2 summarizes studies on the mechanism of PFASs exposure for female reproductive system diseases.

## 5. Nutritional Strategies to Reduce PFASs’ Effects in Female

**Figure 3:**
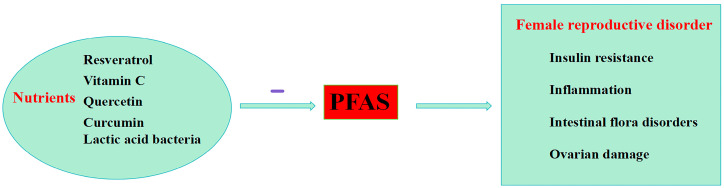
Nutritional supplements mitigate the harmful effects of PFASs on the female reproductive system.

PFASs induce oxidative stress by inhibiting mitochondrial energy production, causing mitochondrial dysfunction [81]. Antioxidants can scavenge excess ROS, helping to maintain the body’s oxidative/antioxidant balance [31]. Emerging research suggests that adopting positive lifestyle modifications, such as antioxidants and anti-inflammatory nutrients, may help reduce the vulnerability to diseases caused by environmental pollutants [82]. The intake of some nutrients can reduce the damage caused by PFASs on the female reproductive system (Figure 3). 

Zhang et al. found that resveratrol supplementation reduces apoptosis by regulating FoxO1 via the SIRT1 and PI3K-AKT pathways. The antioxidant effect of resveratrol was confirmed by examining the RNA levels of mitochondrial electron transport chain-related genes in KGN cells. Resveratrol supplementation significantly increased the RNA levels of these genes, thereby reducing PFOA-induced oxidative stress. Resveratrol also contributed to the amelioration of PFOA-induced ovarian damage, including the protection of ovarian tissue structure and size, the reduction in follicular cell apoptosis, the enhancement of mitochondrial function, and the promotion of follicular development [78]. Nearly half of women diagnosed with PCOS have insulin resistance, which causes hyperinsulinemia [83]. A study from South Korea demonstrated that PFOS and perfluorododecanoic acid (PFDoDA) increase the risk of developing insulin resistance, and that vitamin C supplements protect against this adverse effect. However, this study was based on elderly subjects, and more research is still needed to prove this in those with PCOS [84]. Quercetin and curcumin help reduce the buildup and boost the elimination of PFOA in the body, lowering its bioavailability. They strengthen the intestinal barrier and reduce liver uptake by down-regulating key transport proteins. Quercetin also prevents PFOA from accumulating in the liver by decreasing certain binding proteins [85]. Curcumin, a primary polyphenolic compound found in turmeric, exerts beneficial effects on female reproductive disorders through multiple mechanisms. For PCOS, it reduces inflammation by inhibiting the nuclear factor kappa-light-chain-enhancer of activated B cells (NF-κB)-signaling pathway and lowers androgen levels, aiding follicle maturation and ovulation. In ovarian diseases, curcumin enhances ovarian health by reducing oxidative stress and preventing abnormal cell proliferation. For endometriosis, curcumin alleviates symptoms through its anti-inflammatory properties by inhibiting cyclooxygenase-2 (COX-2) and prostaglandin E2 (PGE2), reduces angiogenesis by downregulating vascular endothelial growth factor (VEGF), and induces apoptosis in endometrial cells by modulating the B-cell lymphoma 2/Bcl-2-associated X protein (Bcl-2/Bax) ratio [83]. Disturbances in intestinal flora play a role in the pathogenesis of PCOS, and PFOS accelerates the development of PCOS by disrupting intestinal environment [86]. Lactic acid bacteria can bind to PFOS and reduce the bioavailability and absorption of PFOS in the body. They also have strong antioxidant properties that help neutralize the oxidative stress caused by PFOS. In addition, LAB improve the intestinal barrier function by up-regulating the production of short-chain fatty acids (SCFAs) and enhancing the expression of tight junction proteins in the intestine [87].

## 6. Conclusions and Perspectives

In conclusion, the cumulative research elucidates a compelling link between PFAS exposure and various detrimental effects on female reproductive health. These include disruptions to the hormonal balance, such as those observed in cases of PCOS and endometriosis, as well as contributions to conditions like POI and DOR. These studies highlight the urgent need for further investigation into the mechanisms by which PFASs exert their effects on the female reproductive system. However, the underlying causes of these diseases are not clear. Most of the current studies are retrospective, and methodological issues limit the inference of causality in the association between exposure to PFASs and the aforementioned diseases in epidemiologic studies. Overall, there is insufficient evidence to establish a causal relationship between exposure to PFASs and diseases of the female reproductive system. Experimental studies and more prospective studies related to human exposure doses should be pursued. In this way, the detrimental effects of PFASs on women’s reproductive health are expected to be improved.

## Figures and Tables

**Table 1:** Epidemiologic evidence of the association between exposure to PFASs and four female reproductive disorders.

Disease	Sample Size (Experimental Group/Controls)	Results	Ref.
PCOS	52/50	Those with PFOA and PFOS serum concentrations in the highest tertile were 6.9- and 5.8-fold more likely to develop PCOS compared to controls, respectively.	[40]
PCOS	30/29	The geometric mean concentration of PFOS was higher in the PCOS group than in the control group.	[41]
PCOS	366/577	Higher concentrations of 6:2 Cl-PFESA, HFPO-DA, PFDoA, and ∑3,4,5 m-PFOS enhanced the risk of PCOS, especially in obese/overweight women.	[42]
PCOS	73/218	Compared to non-PCOS women, PCOS women had higher concentrations of LH and T in their follicular fluid, and PFOA is directly involved in the pathogenesis of PCOS.	[44]
Endometriosis	495/131	PFOA and PFNA raise the risk of endometriosis in women.	[47]
Endometriosis	54/699	Women with endometriosis have more PFOA, PFOS, and PFNA in their blood than women who do not suffer from this condition.	[48]
Endometriosis	157/178	PFOA and PFNA raise the risk of endometriosis in women.	[49]
Endometriosis	240/344	Women with endometriosis have more PFOA, PFOS, and PFNA in their blood than women who are not suffering from this condition.	[50]
POI	120/120	High exposure to PFOA, PFOS, and PFHxS is associated with increased risk of POI. These also impact sex hormone and thyroid hormone levels.	[58]
DOR	64/86	PFUnDA and PFOA were significantly negatively correlated with OSI.	[62]
DOR	185/148	PFHxA was strongly associated with an increased risk of DOR.	[63]
DOR	25/25	The DOR group had higher PFOA exposure than the group with normal ovarian reserve function.	[64]

**Table 2:** In vivo experimental evidence of the effects of PFAS exposure on female reproductive health.

Experimental Subjects	Results	Ref.
Sprague–Dawley female rats	Neonatal and juvenile exposure to PFOA/PFOS in female rats accelerates puberty onset, increases estradiol and LH levels, disrupts estrous cycles, and downregulates Kisspeptin system gene expression.	[67]
Twelve-week-old female ICR mice	PFOS exposure suppresses ERα-induced activation of AVPV–Kisspeptin neurons, leading to prolonged diestrus, reduced corpora lutea, and diminished LH surge, ultimately impairing ovulation in female mice.	[68]
Twelve-week-old female ICR mice	Chronic low-dose PFOS exposure in adult female mice disrupts reproductive endocrine function by reducing the histone acetylation of StAR, leading to decreased estrogen biosynthesis, impaired follicular development, and ovulation failure.	[69]
H295R cell	PFOA and PFOS weakly antagonize ER transactivation, alter steroid hormone levels by inducing aromatase activity, and influence the transcription of genes involved in sex hormone and aldosterone synthesis.	[71]
Adult female mice	PFOA disrupts ovarian function in mice both in vitro and in vivo, causing alterations in hormone levels, steroidogenic gene expression, and folliculogenesis, suggesting a potential risk for premature ovarian failure.	[72]
Porcine theca and granulosa cells	In vitro analysis reveals that PFOS and PFOA disrupt steroidogenic secretion in porcine ovarian cells, inhibiting hormone secretion even under gonadotropic stimulation.	[73]
Mt Shasta strain juvenile rainbow trout	PFAAs exhibit estrogen-like activity in juvenile rainbow trout and bind to estrogen receptors in various species.	[75]
MCF-7 BUS and MVLN cells	Various PFASs exhibit both estrogenic and antiestrogenic effects.	[76]
Six-week-old ICR mice	PFOA exposure impairs follicular development in mice, increases granulosa cell mtROS and apoptosis, and results in reduced follicular reserve.	[78]
Twelve-week-old female ICR mice	Exposure to PFOA in mice leads to the down-regulation of the Kissin–reproductive endocrine system via enhanced PPARα-mediated hepatic FGF21 expression, potentially resulting in prolonged luteal phase and ovulation failure.	[79]

## Data Availability

Data availability is not applicable to this article as no new data were created in this study.

## Abbreviations

The following abbreviations are used in this manuscript:

AMH	Anti-mullerian hormone
ARC	Hypothalamic arcuate nucleus
AVPV	Anteroventral periventricular nucleus
AFC	Antral follicle count
Bcl-2/Bax	B-cell lymphoma 2/Bcl-2-associated X protein
BPA	Bisphenol A
C=F bonds	Carbon-fluorine bonds
COX-2	Cyclooxygenase-2
CYP17A1	17α-hydroxylase
CYP19A1	Cytochrome P450 aromatase
DOR	Diminished ovarian reserve
E2	Estradiol
EDCs	Endocrine-disrupting chemicals
ER	Estrogen receptor
FGF21	Fibroblast growth factor 21
FSH	Follicle-stimulating hormone
FT3	Free triiodothyronine
FT4	Free thyroxine
GenX	Hexafluoropropylene oxide dimer acid
GnRH	Gonadotropin-releasing hormone
HFPO-DA	Hexafluoropropylene oxide dimer acids
HPG axis	Hypothalamic-Pituitary-Gonadal Axis
Kiss1r	Kisspeptin receptor
LH	Luteinizing hormone
MtROS	Mitochondrial ROS
NF-κB	Nuclear factor kappa-light-chain-enhancer of activated B cells
NOR	Normal ovarian reserve
P450scc	Cholesterol side-chain cleavage enzyme
PCBs	Polychlorinated biphenyls
PCOS	Polycystic ovary syndrome
PFCAs	Perfluoroalkyl carboxylic acids
PFASs	Per- and polyfluoroalkyl substances
PFBA	Perfluorobutanoic acid
PFBS	Perfluorobutanesulfonic acid
PFDA	Perfluoroundecanoic acid
PFDoA	Perfluorododecanoic acid
PFDoDA	Perfluorododecanoic acid
PFHxA	Perfluorohexanoic acid
PFHxS	Perfluorohexane sulfonate
PFNA	Perfluorononanoic acid
PFOA	Perfluorooctanoic acid
PFOS	Perfluorooctane sulfonate
PFPeA	Perfluoropentanoic acid
PFUnDA	Perfluoroundecanoic acid
PFHxS	Perfluorohexane sulfonate
PFOS	Perfluorooctane sulfonate
PFSAs	Perfluoroalkane sulfonates
PFPeA	Perfluoropentanoic acid
POI	Premature ovarian insufficiency
P4	Progesterone
PGE2	Prostaglandin E2
PPARs	Peroxisome proliferators-activated receptors
PRL	Prolactin
SCFAs	Short-chain fatty acids
T	Testosterone
T4	Thyroxine
TSH	Thyroid-stimulating hormone
UCA	Urocanic acid
VEGF	Vascular endothelial growth factor
17β-HSD	17β-hydroxysteroid dehydrogenase
3β-HSD	3β-hydroxysteroid dehydrogenase
6:2 Cl-PFESA	6:2 chlorinated polyfluorinated ether sulfonate
